# *Fusobacterium nucleatum* Affects Cell Apoptosis by Regulating Intestinal Flora and Metabolites to Promote the Development of Colorectal Cancer

**DOI:** 10.3389/fmicb.2022.841157

**Published:** 2022-03-18

**Authors:** Tingting Yu, Ling Ji, Liqin Lou, Shiqing Ye, Xiaoting Fang, Chen Li, Feizhao Jiang, Hongchang Gao, Yongliang Lou, Xiang Li

**Affiliations:** ^1^Wenzhou Key Laboratory of Sanitary Microbiology, Key Laboratory of Laboratory Medicine, Ministry of Education, School of Laboratory Medicine and Life Sciences, Wenzhou Medical University, Wenzhou, China; ^2^Colorectal Cancer Research Center, Wenzhou Medical University, Wenzhou, China; ^3^The First Affiliated Hospital of Wenzhou Medical University, Wenzhou, China; ^4^School of Pharmacy, Wenzhou Medical University, Wenzhou, China

**Keywords:** metabolites, *Fusobacterium nucleatum*, colorectal cancer, intestinal flora, cell apoptosis

## Abstract

**Background/Aims:**

Intestinal flora, especially *Fusobacterium nucleatum (Fn)*, can affect the development of colorectal cancer (CRC). In this study, we examined the composition of intestinal flora and their metabolites in the tissues, serum and feces of CRC patients.

**Materials and Methods:**

CRC tissues, adjacent normal colonic tissues, fecal and serum samples were collected from CRC patients who received surgical treatment between January 2018 and January 2020. Fecal and serum samples were collected from healthy individuals for comparison. In addition, fecal samples were collected from BALB/c female mice. SW480, a human CRC cell line, was utilized for *in vitro* studies. The experiments involved ^1^H-NMR-based metabolomics analysis, targeted and untargeted mass spectrometry analysis, and intestinal flora 16S rDNA V4 region sequencing.

**Results:**

The abundance of *Bacteroides* and propionic acid concentration were decreased and that of *Lactobacillus* and lactic acid concentration were increased in CRC tissues. In addition, the abundances of *Ruminococcus*, *Prevotella*, and *Sutterell* were decreased in CRC patients. The levels of leucine and isoleucine were decreased in the serum and tumor tissues of CRC patients. Aspartate, glutamate and glutathione levels were elevated in the tissues of CRC patients only. The serum glutamine, tyrosine, valine, alanine, and histidine levels were decreased significantly. Lactic acid inhibited and propionic acid promoted apoptosis among SW480 CRC cells.

**Conclusion:**

*Fn* affected the apoptosis of CRC cells and promoted the progression of CRC by affecting the distribution of intestinal flora, which altered the concentrations of metabolites such as lactic acid, propionic acid. Intestinal flora could regulate amino acid metabolism.

## Background

Colorectal cancer (CRC) is one of the most common cancers worldwide with more than 1.85 million new cases and 850,000 deaths every year (Torre et al., 2015; Biller and Schrag, 2021). Intestinal microbes have been found to play an important role in CRC development (Rowland, 2009; Wang et al., 2012, 2021; Zhu et al., 2013). The gut microbiota consists of more than 1,000 species of bacteria, and the number of encoded genes within these species is 150 times that of human genome (Qin et al., 2010; Zou et al., 2018).

Microbial colonization is known to impact glucose, amino acid and lactic acid metabolism (Zhou and Fang, 2018). *Lactobacillus* is one of the main intestinal floras producing lactic acid. Additionally, *Lactobacillus* participates in amino acid metabolism via a proteolytic system consisting of proteinases and peptidases to obtain amino acids (Law and Kolstad, 1983). Previous studies have reported that about 15% of *Lactobacillus* strains detected in fermented Asian foods produce glutamic acid (Zareian et al., 2012). *Bacteroides*, a common intestinal flora, ferments complex sugars into many by-products, including short-chain fatty acids (SCFA), such as propionate, formate, acetate, and butyrate. Genome-wide association studies have shown that the proportion of *Bacteroides* in the stool of obese individuals is significantly reduced, and its proportion in stools negatively correlates with serum glutamate levels (Liu et al., 2017). Liu et al. (2017) reported that depletion of species from the *Bacteroides* genus in obese individuals is related to a higher concentration of aromatic amino acids (AAA) and branched chain amino acids (BCAA) such as valine and leucine in the circulation.

Dysregulated cellular apoptosis plays an important role in the development of certain cancers. Bonfili et al. (2017) found that caspase-mediated apoptosis can be induced in HCT116 CRC cells by treatment with a mixture of essential amino acids (EAA) and non-essential amino acids (NEAA). Recent evidence suggests that *Fusobacterium nucleatum* (*Fn*) promotes the formation and development of CRC (Kostic et al., 2013; Gur et al., 2015; Yang et al., 2017). Zhang et al. (2019) showed that the abundance of *Fn* as estimated by quantitative PCR (qPCR) is significantly higher in the tumor tissues and feces samples of CRC patients. In a study by Mima et al. (2016) involving 1,069 CRC patients, the authors found that the higher abundance of *Fn* was significantly associated with shorter survival. However, the exact underlying mechanisms by which *Fn* promotes tumor growth are still unknown.

In the present study, we determined the metabolites in the serum and colonic tissues of CRC patients and the composition of the bacterial flora in their feces. We also explored the impact of *Fn* on CRC by studying the fecal metabolites in mice after oral gavage of *Fn*.

## Materials and Methods

### Study Subjects

Patients with histologically confirmed CRC who underwent surgical treatment at the First Affiliated Hospital of Wenzhou Medical University, Zhejiang, China between June 2018 and January 2020 were prospectively enrolled in this study. The study was approved by the Ethics Committee of the First Affiliated Hospital of Wenzhou Medical University. Written informed consent was obtained from all participants (patients and healthy subjects) before participation in the study.

The samples collected from the patients included resected CRC tissues, colonic tissues adjacent to the tumor, feces, and serum. Tumor staging was done according to the American Joint Committee on Cancer (AJCC), 8th edition. All fresh resected tissues and fecal samples were taken to the laboratory within 30 min and stored at −80°C within 2 h. Forty-four fecal samples from CRC patients were collected for the study. Additionally, in July 2019, 61 fecal samples from 61 healthy subjects were collected for the control group. The 61 healthy subjects, aged 40–62 years, were selected as controls during a routine physical examination in the First Affiliated Hospital of Wenzhou Medical University, and none of them had had a gastrointestinal tract disorder or taken any antibiotics in the previous 3 months before sample collection. Total fecal DNA was obtained using an extraction kit (Hangzhou Guhe Biotechnology Co., Ltd., Hangzhou, China) according to the manufacturer’s protocol. The V4 region of the bacterial 16S rDNA marker gene (16S V4) was polymerase chain reaction (PCR) amplified and sequenced by Hangzhou Guhe Biotechnology Co., Ltd. The forward primer for PCR amplification of the V4 region of the bacterial 16S rRNA gene was 5′-GTGCCAGCMGCCGCGGTAA-3′ and the reverse primer was 5′-GGACTACHVGGGTWTCTAAT-3′.

### ^1^H-NMR-Based Metabolomics Analysis

The fresh CRC tissues were carefully dissected to obtain 200–500 mg of tissue samples and suspended in a mixture of methanol (4 mL/g of tissue) and double distilled water (0.85 mL/g of tissue). The suspension was centrifuged with 20 strokes at 800 rpm, and 50% chloroform (2 mL/g of tissue) was added, followed by repeat homogenization. The samples were centrifuged at 1,000 rpm for 30 min at 4°C. The water layer of each specimen was separated and evaporated to dryness under a stream of nitrogen. The residue was mixed with 580 μL D_2_O containing 30 μM phosphate-buffered saline (PBS; pH = 7.4) and 0.01 mg/mL sodium-3-(trimethylsilyl)-2,2,3,3-tetradeuteriopropionate (TSP) as an internal standard (δ0.0). After centrifugation at 12,000 rpm for 5 min, the supernatant was transferred to a 5-mm NMR tube for NMR spectroscopy. The ^1^H NMR spectra of tumor tissue were obtained on a Bruker AVANCE III 600 MHz and equipped with a triple resonance probe at 298 K. Moreover, typical acquisition parameters were set as follows: scans = 256; spectral width = 12,000 Hz; data points = 64 K; relaxation delay = 6 s; acquisition time = 2.65 s per scan.

Fasting blood sample was collected in a 5 mL vacutainer tube containing the chelating agent ethylene diamine tetraacetic acid (EDTA) and centrifuged at 1,500 g for 15 min at 4°C. Serum was collected and stored at −80°C until analysis. ^1^H NMR spectra were recorded using a Bruker AVANCE III 600 MHz NMR spectrometer with a 5-mm TXI probe (Bruker BioSpin, Rheinstetten, Germany) at 37°C. Serum samples were thawed at 4°C and vortexed for 10 s using a vortex-genie (Thermo Fisher Scientific Industries). Then 200 μL of serum sample was drawn into an Eppendorf tube and mixed with 400 μL of 0.2 mol/L phosphate buffer. The mixture was centrifuged at 10,000 g for 10 min at 4°C, and 500 μL of supernatant was transferred and mixed with 100 μL of D_2_O containing 0.5% TSP in a 5 mm NMR tube for metabolomics analysis. ^1^H NMR spectra were acquired using the CPMG pulse sequence with a fixed receiver-gain value and the main parameters were set as follows: relaxation delay, 4 s; acquisition time, 1.64 s/scan; data points, 32K; spectral width, 10,000 Hz; exponential line-broadening function, 0.3 Hz.

Subsequently, all spectra were aligned using the “icoshift” procedure in MATLAB (R2012a, The Mathworks Inc.).

### Mass Spectrometry for Metabolism

Fecal samples collected from the mice and tissues collected from the CRC patients were mixed with pre-cooled methanol/acetonitrile/water solution in the ratio of 2:2:1 (v/v), followed by vortex mixing, low-temperature ultrasonic processing for 30 min, freezing at −24°C for 10 min, and then centrifugation at 14,000 rpm for 20 min at 4°C. Later, the supernatant was extracted and dried in vacuum. Subsequently, 100 μL of acetonitrile aqueous solution (acetonitrile: water = 1:1, v/v) was added for mass spectrometry, followed by vortex mixing and centrifugation at 14,000 rpm at 4°C for 15 min. Finally, the samples were sent with clear liquid for analysis by Shanghai Applied Protein Technology Co., Ltd. (Shanghai, China). Mouse feces and human tissues were detected by Agilent 7890B/7000D GC-MS (Agilent, United States) for targeted detection of SCFA. In addition, mouse feces were also tested for untargeted metabolites by UHPLC-Q-TOF MS (Agilent, United States; AB SCIEX, United States).

### *Fusobacterium nucleatum* Strain and Culture

The *Fn* strain (**ATCC** 25586) used in this study was purchased from the American Type Culture Collection (Manassas, VA, United States). The *Fn* strain was cultured in Columbia blood agar supplemented with 5% defibrinated sheep blood, 5 g/mL hemin, and 1 μg/mL vitamin K1 (Sigma-Aldrich, St. Louis, MO, United States) and incubated in a 37°C anaerobic glove box with 5% CO_2_, 10% H_2_, and 85% N_2_.

### Animal Experiments

Thirty-six BALB/c female mice (6–8 weeks old) were purchased for this animal study (Beijing Vital River Laboratory Animal Technology Co., Ltd.). The study involving the experimental animals was approved by the Institutional Animal Committee of Wenzhou Medical University. The mice were divided into three groups: control group, *Fn* + NaCl (Beyotime Institute of Biotechnology) group, and *Fn* + azoxymethane (AOM; 12 mg kg^–1^; Sigma-Aldrich, United States) group, with 12, 10, and 14 mice in each group, respectively. Each mouse weighed about 20 g. After 1 week of acclimatization, AOM/0.9% NaCl was injected intraperitoneally once. One week later, the mice were given the *Fn*/0.9% NaCl by oral gavage, once a day, for 5 consecutive days. One week after stopping the intervention, mice were given the intraperitoneal injection of AOM/0.9% NaCl. One week after the second AOM/0.9% NaCl injection, *Fn* was given via another oral gavage for 5 days. Lastly, the mice were given water and normal food until the end of the experiment (81st day) (Supplementary Figure 1). The concentration of the intraperitoneal AOM injection was 10 mg/kg. The amount of *Fn* used was 1 × 10^8^ per mouse (OD_600 nm_ = 0.1) with 200 μL 0.9% NaCl.

### SW480 Culture and Treatment

SW480 cell line was obtained from ATCC and cultured in Dulbecco’s Modified Eagle Medium (DMEM) supplemented with 10% fetal bovine serum (FBS) (Invitrogen, Waltham, MA, United States), 100 mg/mL streptomycin (Sangon, Shanghai, China) and 100 U/mL penicillin G (Sangon, Shanghai, China) at 37°C in an incubator containing 5% CO_2_. SW480 was cultured in a petri dish. Next, the culture medium of SW480 cells that had grown adherently was discarded and washed with PBS three times, and then fresh culture medium and lactic acid (L1750, Sigma-Aldrich, United States) or propionic acid (P5436, Sigma-Aldrich, United States) were added to culture for 48 h for subsequent experiments. The experiment was repeated thrice.

### Western Blot Analysis

WB analysis was conducted to determine the protein expression in SW480 cells. In brief, total protein was extracted and concentration was measured using the BCA protein assay. Total protein (20 μg) was initially separated on 12% sodium dodecyl sulfate polyacrylamide gel electrophoresis and transferred to a nitrocellulose membrane. After blocking in 5% milk, membranes were incubated with specific primary antibodies (1:1,000 dilution) including Cleaved-PARP (Diagbio, Hangzhou, China), Cleaved-caspase3 (Diagbio, Hangzhou, China), Bcl-2 (Diagbio, Hangzhou, China), Bax (Diagbio, Hangzhou, China), and β-actin (Abways, Beijing, China), at 4°C, overnight. Membranes were subsequently incubated with HRP-conjugated secondary antibody (Beyotime Institute of Biotechnology). Levels of protein were quantified using ImageJ 1.43u/Java 1.6.0-10 from the National Institutes of Health (Bethesda, MD, United States).

### Statistical Analysis

The R package (ropls) was used to analyze the data after normalization to the total peak intensity. Multivariate data analysis, including orthogonal partial least-squares discriminant analysis (OPLS-DA), was performed. Sevenfold cross-validation and response permutation testing was used to evaluate the robustness of the model. The variable importance in the projection (VIP) value for each variable in the OPLS-DA model was calculated to assess its contribution to the metabolite levels. Those with a VIP value > 1 were further subjected to Student’s *t*-test at the univariate level to measure the significance for each metabolite. *P*-values less than 0.05 were considered as statistically significant.

## Results

### Composition of the Intestinal Flora of the Healthy Subjects and Colorectal Cancer Patients

**FIGURE 1:**
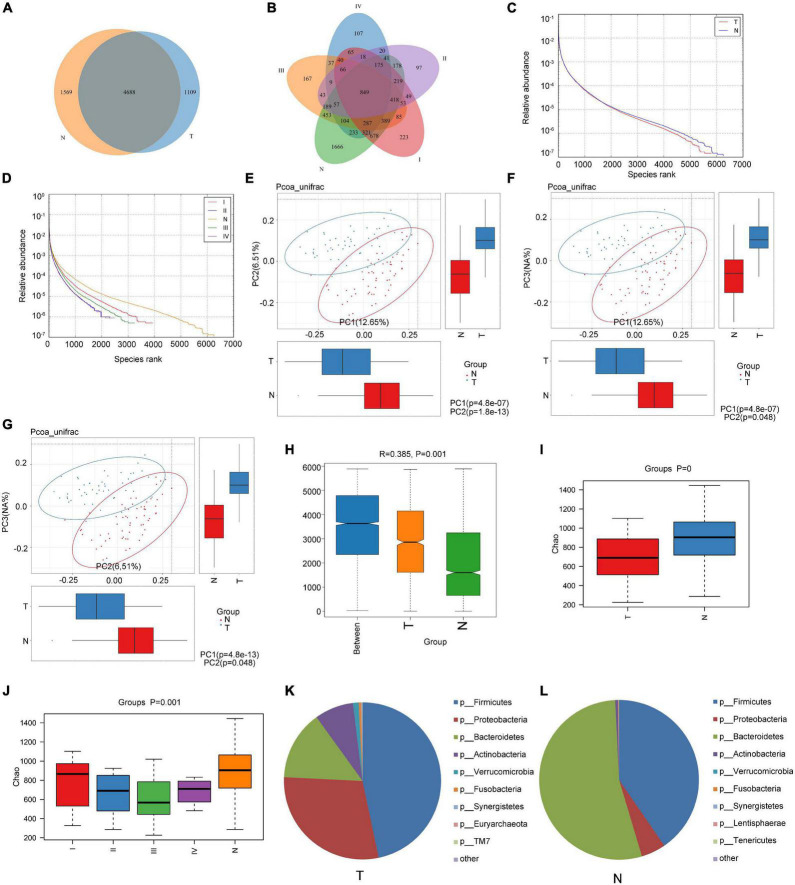
Comparison of the composition of intestinal flora between healthy subjects and CRC patients. **(A,B)** Venn diagrams according to the different grouping methods; **(C,D)** rank abundance curve according to the different grouping methods; **(E–G)** principal Co-ordinates Analysis (PCoA) with unweighted Unifrac distance; **(H)** use of ANOSIM to analyze differences between the groups and within the groups; **(I,J)** Chao1 index analysis according to the different grouping methods; and **(K,L)** analysis of the composition of the intestinal flora of cancer patients and healthy subjects at the phylum level.

Fecal samples were collected from 44 patients (T group) having CRC of different stages and 61 samples from healthy individuals (N group). The relevant demographic and clinical details are presented in Table 1. The fecal samples were used for high-throughput sequencing and sequence extraction, splicing, and optimization (the 16s sequencing accession numbers are PRJNA799322, PRJNA799208, and PRJNA799246). All samples were selected with the smallest number of sequences in the sample. By default, a 16S rRNA sequence similarity higher than 97% can be defined as an operational taxonomic unit (OTU). According to the results of OTU clustering, a Venn diagram was drawn and the numbers of common and unique OTUs between different groups were compared. As shown in Figure 1A, there were 1569 OTUs in the N group, 1109 OTUs in the T group, and 4688 OTUs in both groups. According to the TNM stage of CRC patients, there were 1,666, 223, 97, 167, 107 unique OTUs in the N, stage I, II, III, and IV groups, respectively (Figure 1B). The rank abundance curve showed that the abundance in the N group was higher than that in the T group, and the difference in abundance between the OTUs was smaller in the N group than that in the T group (Figure 1C). Also, the abundance of fecal flora in patients with stage II and IV CRC was similar and the smallest among those of patients with different stages of CRC, but the difference in the abundance between OTUs was the largest (Figure 1D).

**TABLE 1:** Clinical characteristic of all individuals.

	Healthy volunteers	CRC patients
N	61	44
Age (Median, Range)	52, 40–62	60.5, 40–84
Male/Female	32/29	27/17
**CRC stage**		
Stage I		14
Stage II		8
Stage III		14
Stage IV		8

Using the unweighted Unifrac distance to perform PCoA analysis, differences between groups were found (Figures 1E–G). ANOSIM indicated that the difference between groups was significantly greater than the difference within groups (*R* = 0.385 > 0 and *P* = 0.001; Figure 1H). The Chao1 index showed that the richness of the communities in the sample was different between the N and T groups, and between N and the patient groups for each stage (Figures 1I,J). However, the Shannon and Simpson indexes reflecting the diversity of the community did not differ significantly (data not shown).

The structural analysis results for the intestinal flora in the T group and N group at the phylum level are shown in Figures 1K,L. In the T group, the top six phyla were *Firmicutes*, *Bacteroidetes*, *Proteobacteria*, *Actinobacteria*, *Verrucobacteria, and Fusobacteria*, and their proportions were 46.68, 29.07, 14.29, 7.99, 1.18, and 0.47, respectively, accounting for 99.69% of the total quantity of microorganisms in the sample. In the N group, the top 6 phyla were *Bacteroidetes*, *Firmicutes*, *Proteobacteria*, *Actinobacteria*, *Fusobacteria*, and *Verrucomicrobia*, and their proportions were 53.84, 40.41, 4.97, 0.56, 0.13, and 0.03%, respectively, accounting for 99.94% of the total microorganisms in the sample. At the phylum level, the relative abundances of *Bacteroidetes*, *Proteobacteria*, and *Actinobacteria* were significantly different between healthy subjects and CRC patients (*P* < 0.05). Our analysis found that despite the unevenness within the group tested, fecal microbiomes did not differ significantly by gender (*P* > 0.05; Supplementary Figure 2A).

### Intestinal Flora Regulates Cell Apoptosis by Affecting Metabolism

**FIGURE 2:**
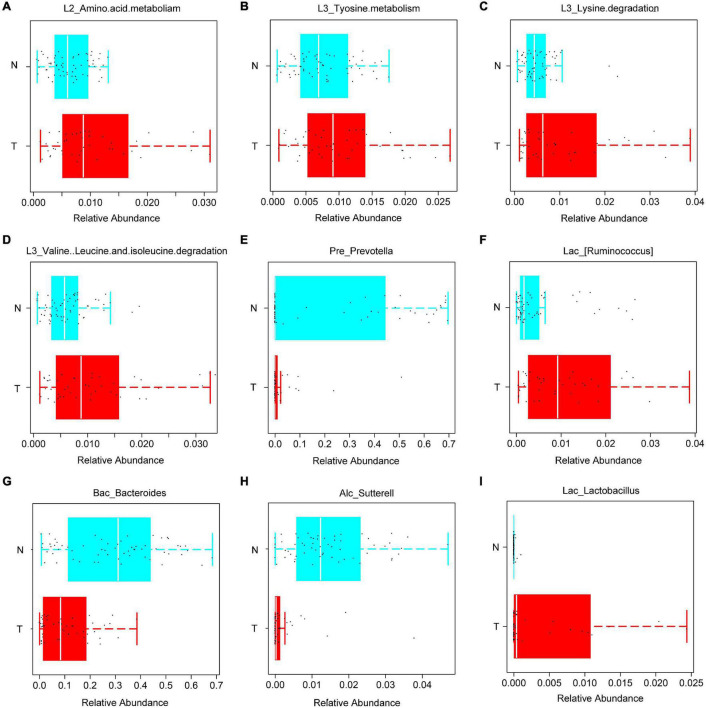
KEGG functions and related bacteria in the CRC group (T) and healthy group (N). **(A–D)** The relative abundances of genera related to different KEGG functions in T and N groups; **(E–I)** bacteria with statistical difference between the T and N groups.

**FIGURE 3:**
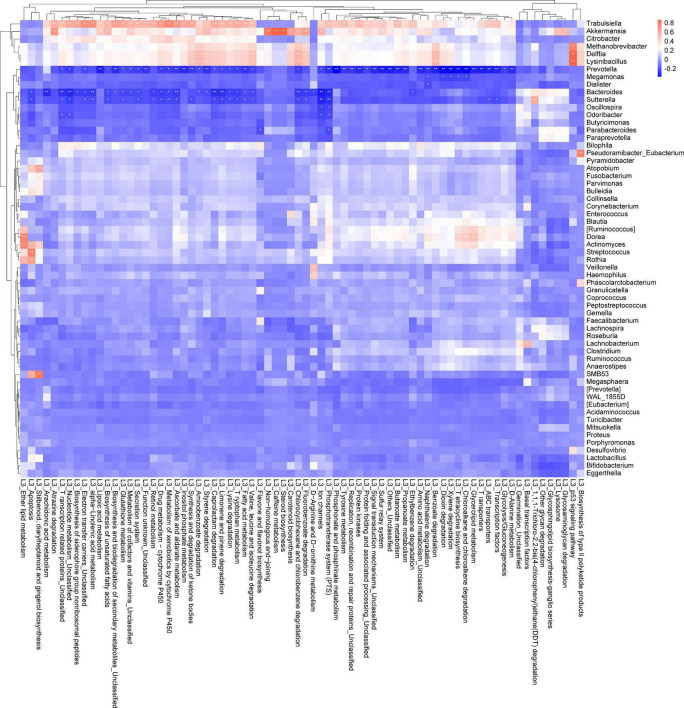
Correlation analysis heat map of bacteria and KEGG pathways.

The abundance of bacteria involved in the metabolism of amino acids such as tyrosine metabolism, lysine, valine, leucine, and isoleucine was increased in the T group (Figures 2A–D). Based on the PICRUSt function prediction of the 16S rDNA sequence, differences in the abundances in functional genes of KEGG pathways at different levels (1∼3) were observed between the T group and N group. Based on this, a heat map was made at the genus level to examine the role of the genera in the different KEGG pathways. Among them, *Prevotella* and (*Ruminococcus*) were found to be involved in amino acid and tyrosine metabolism (Figure 3). Additionally, *Prevotella* played an important role in lysine, valine, leucine, and isoleucine degradation (Figure 3). In the stool sequencing of the T group, we found that the content of *Prevotella* was decreased and that of (*Ruminococcus*) was increased compared with the contents for the N group (Figures 2E,F). In addition, *Bacteroides* and *Sutterel* were found to play roles in the biosynthesis of unsaturated fatty acids, fatty acid metabolism, glutathione metabolism, and lysine, valine, leucine and isoleucine degradation (Figure 3). Notably, *Bacteroides*, *Sutterel* and *Lactobacillus* were all related to the apoptotic pathway. Also, the abundances of *Bacteroides* and *Sutterel* were decreased in the T group, while the abundance of *Lactobacillus* was increased in the T group (Figures 2G–I, 3).

**FIGURE 4:**
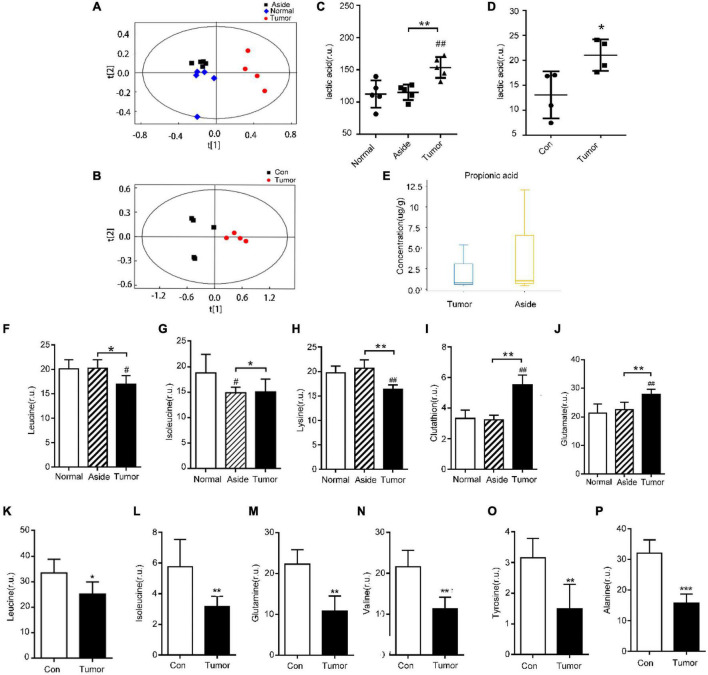
Detection of metabolites in the tissues and serum of CRC patients and healthy people. **(A,B)** Principal component analysis (PCA) of metabolites in tissues and serum of CRC patients, respectively; **(C,D)** detection of lactic acid in tissues and serum of CRC patients, respectively; **(E)** propionic acid in tissues of CRC patients; **(F–J)** differential metabolites in various tissues of CRC patients with NMR, ^#^*P* < 0.05, ^##^*P* < 0.01, *P < 0.05, **P < 0.01; and **(K–P)** differential metabolites in serum of CRC patients and healthy individuals, *P < 0.05, **P < 0.01, ***P < 0.001.

We also collected cancer tissues and adjacent normal colonic tissues from 5 CRC patients, and serum samples from 4 CRC patients and 4 healthy subjects for NMR detection. In the principal component analysis scoring chart (PCOA), we saw a separation trend between the serum of T and N groups, as well as a separation trend between the cancer tissues, the adjacent colonic tissues, and the normal tissues of the CRC patients (Figures 4A,B). Lactic acid as the main metabolite of *Lactobacillus* was found to be increased in both the serum and tumor tissues (Figures 4C,D). The results of mass spectrometry of tumor tissues from CRC patients showed that the content of propionic acid, the main metabolite of *Bacteroides*, was decreased (Figure 4E). Interestingly, *Lactobacillus* showed increased abundance in the feces of CRC patients relative to controls, while *Bacteroides* showed decreased abundance (Figures 2G,I). In addition, the levels of leucine and isoleucine in tumor tissues were significantly lower than those in the adjacent colonic tissues. Correspondingly, the levels of leucine and isoleucine in the serum of CRC patients were lower than those in healthy subjects (Figures 4F,G,K,L). Also, the level of lysine in CRC tissues was decreased (Figure 4H), while the serum levels of valine, histidine, alanine, and tyrosine in the CRC patients were significantly decreased (Figures 4N–P and Supplementary Figure 2B). Notably, the glutamine content in the serum of CRC patients was decreased, while that in the tumor tissues was increased (Figures 4J,M). In addition, the results showed that the levels of aspartic acid and glutathione in CRC tissues were increased (Figure 4I and Supplementary Figure 2D).

Based on the above results, we believe that the intestinal flora plays an important role in the regulation of cell apoptosis by affecting propionic acid and lactic acid metabolism. Amino acid metabolism could also be regulated by the intestinal flora.

### *Fusobacterium nucleatum* Affects Cell Apoptosis by Regulating Intestinal Flora and Associated Metabolites

**FIGURE 5:**
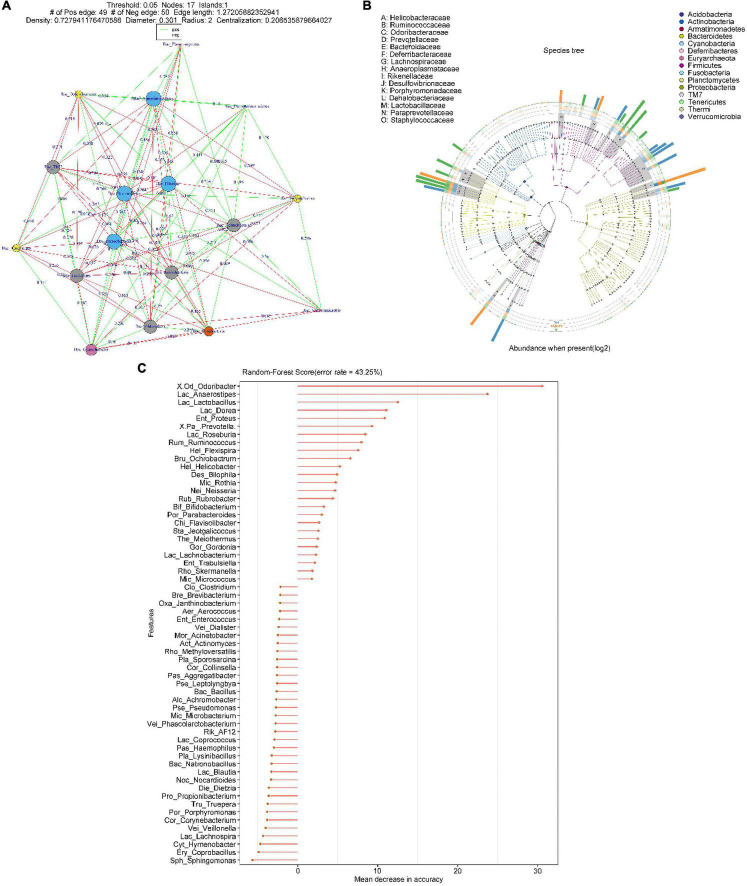
Sequencing analysis of the fecal samples from mice given *Fn* gavage. **(A)** The relationship between microorganisms at the phylum level; **(B)** distribution map of sample community of species evolutionary tree; and **(C)** species importance point map.

**FIGURE 6:**
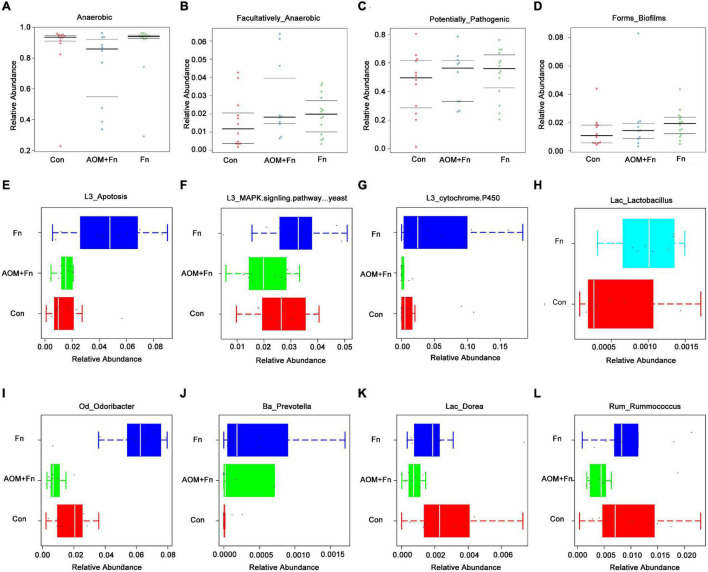
Sequencing analysis of the fecal samples from mice given *Fn* gavage. **(A–G)** The relative abundances of genera related to different KEGG functions in the *Fn* group and control group; **(H)** distribution of *Lactobacillus* in the *Fn* group and control group; **(I–L)** bacteria with statistical difference in expression between the *Fn* group, *Fn* + AOM group and control group.

To explore the effect of oral gavage with *Fn* on the intestinal flora and the intestinal metabolites of mice, we collected feces of a control (Con) group, a *Fn* group and a *Fn* + AOM group of mice for 16S rDNA sequencing and mass spectrometry detection. In the *Fn* group, the top three phyla were *Firmicutes*, *Bacteroidetes*, and *Proteobacteria*, accounting for 40.50, 37.59, and 19.93%, respectively, whereas in the control group, these phyla accounted for 43.68, 27.71, and 24.88%, respectively. The correlation analysis of the microbes at the phylum level showed that the abundance of *Fusobacteria* was positively correlated with the abundance of *Gemmatimonadetes* and negatively correlated with the abundance of *Euryarchaeota* and *Tenericutes* (Figure 5A). *Firmicutes* and *Bacteroidetes* were more abundant in the feces. *Firmicutes* was positively correlated with *Planctomycetes* and *Gemmatimonadetes*, but negatively correlated with *Verrucomicrobia*. Additionally, *Bacteroidetes* was positively correlated with *Actinobacteria* and *Armatimonadetes* (Figure 5A). We used GraPhlan combined with OTU tables to display the results of OTU species annotations of all samples (Figure 5B). Bacteria were mainly distributed in *Firmicutes*, *Bacteroidetes*, and *Proteobacteria*. For *Firmicutes*, the flora of *Ruminococcaceae* and *Lactobacillaceae* were mainly distributed in the *Fn* group. The flora of *Lachnospiraceae*, *Dehalobacteriaceae*, and *Staphylococcaceae* were mainly distributed in the Con group. For *Bacteroidetes*, the flora of *Odoribacteraceae*, *Bacteroidaceae*, *Prevotellaceae*, *Paraprevotellaceae*, and *Porphyromonadaceae* were mainly distributed in the *Fn* group, and *Rikenellaceae* was mainly distributed in the Con group. The random forest algorithm was used to draw a point map of species importance, and the results showed that *Odoribacter*, *Anaerostipes*, *Lactobacillus*, *Dorea*, *Proteus*, *Prevotella*, *Roseburia*, and *Ruminococcus* played major roles in the grouping effect (Figure 5C). The comparison of phenotypic classification based on BugBase showed that the *Fn* + AOM group had the least anaerobic bacteria, and the *Fn* + AOM group and *Fn* group had higher facultative bacteria contents than the Con group (Figures 6A,B). At the same time, in the *Fn* + AOM group and *Fn* group, the abundance of potentially pathogenic bacteria was increased (Figure 6C). In addition, the ability for biofilm formation improved gradually in the Con, *Fn* + AOM, and *Fn* groups (Figure 6D).

**FIGURE 7:**
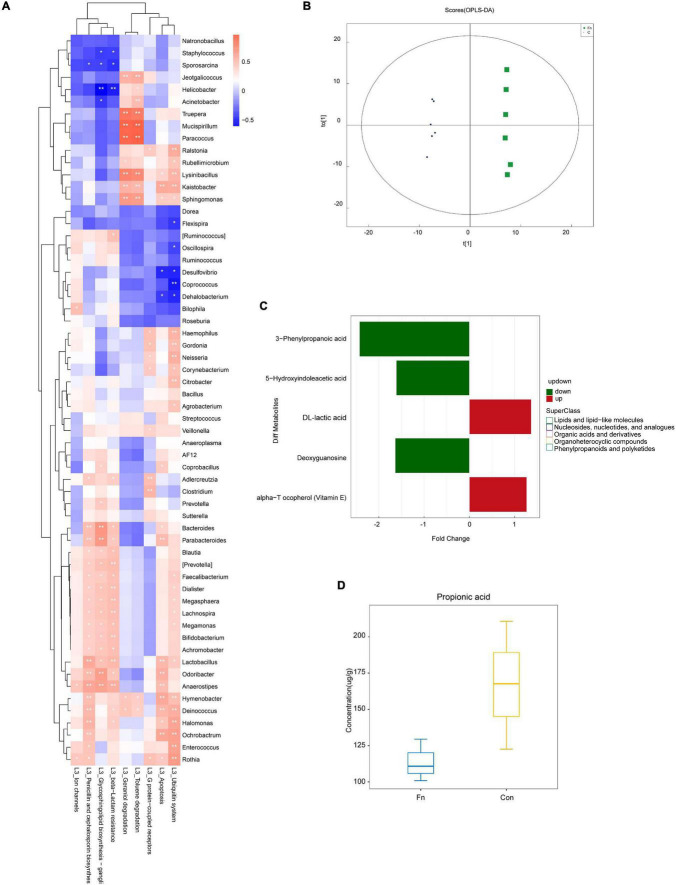
KEGG pathway and metabolite analysis of the fecal samples from mice given *Fn* gavage. **(A)** Correlation analysis heat map of bacteria and KEGG pathways in Con and *Fn* groups. **(B)** OPLS-DA showed the stability of the template; **(C)** differential metabolites identified with non-targeted mass spectrometry; **(D)** propionic acid identified with targeted mass spectrometry.

The results of KEGG pathway analysis showed that the abundance of the flora associated with the apoptosis pathway was increased in the *Fn* group (Figure 6E). Compared with the Con group, the flora related to the MAPK signaling pathway was enriched in the *Fn* group, but decreased in the *Fn* + AOM group, which was similar to the distribution of the cytochrome p450-related flora (Figures 6F,G). These series of results seem to point to an apoptotic pathway related to mitochondria. The analysis of the abundance of fecal flora showed that compared with the Con group, the abundance of *Odoribacter*, *Dorea*, and *Rummococcus* were increased in the Fn group but decreased in the *Fn* + AOM group. *Prevotella* increased significantly in the Fn group compared to the other two groups (Figures 6I–L). In addition, the comparative analysis of *Fn* group and Con group showed that the abundance of *Lactobacillus* was increased significantly in the *Fn* group, but no difference was found in the three-group comparative analysis of the *Fn* group, Con group and *Fn* + AOM group (data not shown; Figure 6H). At the same time, the correlation heat map of KEGG pathways and flora showed that *Lactobacillus*, *Odoribacter*, and *Bacteroides* were related to the apoptosis pathway in the two-group comparative analysis of the *Fn* group and Con group (Figure 7A). The above results showed that *Lactobacillus* and *Bacteroides* play an important role in the composition of the intestinal flora of mice given *Fn* gavage. OPLS-DA indicated that the model was stable (Figure 7B). VIP > 1 and *P*-value < 0.05 obtained using the OPLS-DA model were the screening criteria used to label the difference in the metabolites as significant. As shown in Figure 7C, in the *Fn* group, 3-phenylpropanoic acid, 5-hydroxyindoleacetic acid and deoxyguanosine were decreased. However, alpha-tocopherol (vitamin E) was increased (Figure 7C). D-lactic acid, the main metabolite of *Lactobacillus*, was significantly increased in the *Fn* group (Figure 7C). In addition, propionic acid showed a downtrend in the *Fn* group compared to the Con group (Figure 7D).

Based on the above results, we believe that in mice given *Fn* gavage, *Bacteroides* and *Lactobacillus* in the intestinal flora can affect cell apoptosis by regulating lactic acid and propionic acid metabolism.

### Effects of Lactic Acid and Propionic Acid on the Apoptosis of SW480 Colorectal Cancer Cells

**FIGURE 8:**
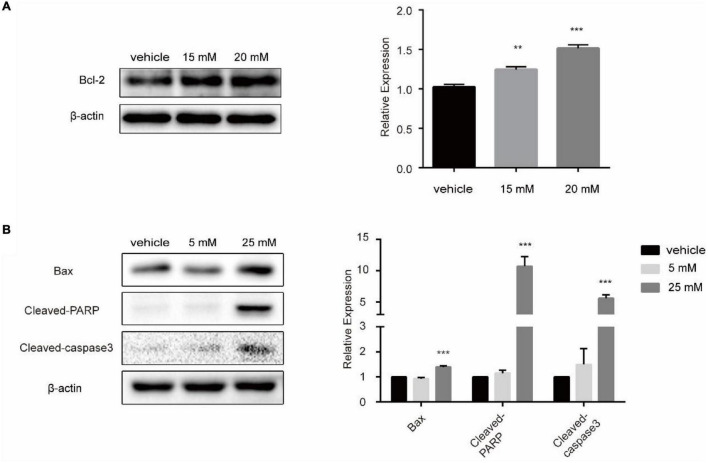
The influence of lactic acid and propionic acid on the apoptosis of SW480 cells. **(A)** Lactic acid inhibited the apoptosis of SW480 colorectal cancer cells. **(B)** Propionic acid promoted the apoptosis of the SW480 colorectal cancer cells. **P < 0.01, ***P < 0.001 vs. control.

To confirm the influence of lactic acid and propionic acid on the apoptosis pathway at the cellular level, we treated SW480 CRC cells with them to detect the expression of related proteins in the apoptosis pathway. We cultured SW480 cells with 15.20 mM lactic acid in a sugar-free environment for 48 h. Western Blot analysis showed that the expression of anti-apoptotic protein Bcl-2 was increased (Figure 8A). After treating SW480 cells with different concentrations of propionic acid, western blot analysis showed that Bax, Cleaved-Parp and Cleaved-caspase 3 expression increased significantly after treating the cells with 25 mM propionic acid for 48 h. There was an upward trend in protein expression after treatment with 5 mM propionic acid, but the difference was not statistically significant (Figure 8B). Based on these findings, we concluded that lactic acid, a metabolite of *Lactobacillus*, inhibited the apoptosis of SW480 cells, while propionic acid, a metabolite of *Bacteroides*, promoted apoptosis among SW480 cells.

## Discussion

The results of this study indicate that the intestinal flora plays an important role in the occurrence and progression of CRC. We found that the diversity of the intestinal flora was decreased in the feces of CRC patients (Figure 1). In addition, *Fusobacterium* was found to be associated with the apoptotic pathway (Figure 3). These findings indicated that *Fn* could play an important role in the occurrence and development of CRC. We have confirmed in our previous work that at the cellular level, *Fn* promotes the progression of CRC through the Cdk5-activated Wnt/β-catenin signaling (Li et al., 2020). The experiments of the present study showed that the intestinal flora of mice given *Fn* gavage mainly included *Firmicutes*, *Bacteroidetes*, and *Proteobacteria* (Figure 5B). In addition, the abundances of *Lactobacillaceae*, *Odoribacteraceae*, *Bacteroidaceae*, *Prevotellaceae*, and *Paraprevotellaceae* were increased in the *Fn* group (Figure 5B). Interestingly, our results showed a decreasing abundance of *Bacteroidetes* and a decreasing abundance of *Bacteroides* in fecal specimens from CRC patients (Figures 1K,L, 2G). In the fecal samples of mice in the *Fn* group, the abundance of *Bacteroidetes* was decreased (*P* < 0.05, data not shown) and the abundance of *Bacteroidaceae* was increased (Figure 5B). However, the difference in the abundance of *Bacteroides* was not statistically significant (*P* > 0.05, data not shown). Previous studies have found that the richness and diversity index of *Bacteroides* differ significantly between the mouse and human gut (Li, 2011). Species differences may contribute to inconsistent *Bacteroides* results in mouse and human specimens. Additionally, certain bacterial genera in these families such as *Lactobacillus*, *Bacteroides*, *Odoribacter*, and *Parabacteroides* were related to the apoptosis pathway, and the abundances of *Lactobacillus* and *Odoribacter* were increased in the *Fn* group (Figures 6H,I, 7A). The KEGG pathway analysis showed that the abundance of anaerobic, facultative and potentially pathogenic bacteria was increased in the *Fn* group (Figures 6A–C). The capability for biofilm formation improved gradually in the Con, *Fn* + AOM, and *Fn* groups (Figure 6D). The results indicated that *Fn* could regulate the composition and related functions of the intestinal flora to promote the development of CRC.

Jacobson et al. (2018) found that propionate produced by *Bacteroides* directly inhibits *Salmonella enterica serovar Typhimurium* growth *in vitro* by disrupting intracellular pH homeostasis and also limits their fecal shedding *in vivo*. We observed a downward trend in the propionic acid level in cancer tissues of CRC patients, and the abundance of propionic acid-producing *Bacteroides* was decreased significantly (Figures 2G, 4E). The KEGG pathway analysis showed that *Bacteroides* were related to the apoptotic pathway (Figure 3). The same findings were noted in the stool of mice given *Fn* gavage. In *these* mice, the KEGG pathway analysis of propionic acid-producing *Bacteroides* was found to be related to the apoptosis pathway (Figure 7A). The content of propionic acid was decreased in the feces of mice given *Fn* gavage but not statistically significant due to the limited number of mice (Figure 7D). We also found that propionic acid promoted the apoptosis of SW480 CRC cells (Figure 8B).

Chan et al. (2009) found that the content of lactic acid was increased in cancer tissues with the tissue metabolism profile. In our study, we also found that the content of lactic acid in the serum and cancer tissues of CRC patients was increased (Figures 4C,D), and the abundance of lactic acid-producing *Lactobacillus* was increased significantly. The KEGG pathway analysis showed that *Lactobacillus* was related to the apoptotic pathway (Figures 2I, 3). Similarly, the KEGG pathway analysis of lactic acid-producing *Lactobacillus* was also found to be related to the apoptosis pathway in mice given *Fn* gavage (Figures 7A,C). It is worth noting that when compared with that in the control group, the abundance of *Lactobacillus* in the *Fn* group was significantly increased (*P* < 0.05). When the *Fn*, *Fn* + AOM and control groups were compared, no statistical difference was found in the distribution of *Lactobacillus* (data not shown; Figure 6H). The *in vitro* experiment in this study found that lactic acid inhibited the apoptosis of SW480 cells (Figure 8A).

In addition, the intestinal flora related to the MAPK and cytochrome p450 pathways were enriched in the *Fn* group. Among them, *Odoribacter* and *Rummoccus* were related to the MAPK pathway, while *Bacteroides* was related to the cytochrome p450 pathway (Supplementary Figure 2C and Figures 6I,L). The mammalian p450s are all membrane-bound (mostly bound to the endoplasmic reticulum membrane, but some to the mitochondrial membrane) (Guengerich, 2019). The MAPK pathway plays an important role in regulating apoptosis related to growth factors in CRC (Fang and Richardson, 2005). Whether *Fn* regulates mitochondrial function by regulating the MAPK signaling pathways and cytochrome p450 and then affects cell apoptosis remains to be explored. Therefore, we will continue to do related research in the future, including western blot analyses.

We believe that *Fn* promotes the progression of CRC by affecting the distribution of *Bacteroides* and *Lactobacillus*. *Fn* promotes interspecies adhesion and biofilm formation through outer membrane protein RadD (Kaplan et al., 2009). *Fn* can occupy the binding site of epithelial cells through adhesion factors, thereby preventing other bacteria from attaching and invading epithelial cells, and selectively inhibiting other dominant flora (Kim et al., 2008; Guo et al., 2018). This may prevent the *Bacteroidetes* from attaching to epithelial cells and lead to a decrease in their abundance. At the same time, *Fn* also showed weak antagonistic activity against *Lactobacillus*, an important group in the small intestinal microbiota (Guo et al., 2018). However, the interaction between *Fn*, *Bacteroidetes* and *Lactobacillus* needs to be further explored. In addition, invasive adhesion molecules of *Fn* can promote bacterial aggregation and biofilm formation (Gursoy et al., 2010). Ding et al. (2021) found that heat-killed *Lactobacillus acidophilus* can co-aggregate with *Fn*, the bridging bacteria of oral biofilm, and inhibit the adhesion and invasion of *Fn*, leading to a subsequent elimination of pro-inflammatory cytokine production in oral epithelial cells. However, the role of *Lactobacillus* in this process requires further exploration (Ding et al., 2021). We also believe that *Bacteroides* and *Lactobacillus* can affect the apoptosis of CRC cells through metabolites such as lactic acid, propionic acid, and amino acids.

In a study by Li et al. (2019) involving the serum samples of 120 healthy volunteers, 120 multiple sclerosis patients, and 120 age- and gender-matched CRC patients, the contents of tyrosine and Glu-Leu dipeptide were decreased in CRC patients, which indicated that the combination of Glu-Leu and tyrosine in serum might be useful as a new biomarker for the early diagnosis of CRC. Similarly, in the current study, the serum tyrosine levels of CRC patients were significantly decreased (Figure 4O). These findings might be due to higher utilization of the amino acids by CRC cells to maintain the rapid cell proliferation (Snell et al., 1988). Also, research has shown that hypoxia-responsive miRNAs might be involved in the targeted regulation of β-alanine, valine, leucine, and isoleucine metabolism (Nijhuis et al., 2017). Our results showed that alanine and valine were decreased in serum of CRC patients (Figures 4N,P). Also, leucine and isoleucine were decreased in serum and tissues of CRC patients (Figures 4F,G,K,L). Whether the intestinal flora regulates the amino acid metabolism through hypoxia-responsive miRNA remains to be studied. The results showed that the content of glutathione in CRC tissues was increased (Figure 4I). Excessive glutathione has been reported to be associated with tumor progression and distant metastasis (Bansal and Simon, 2018). In this study, we found that *Bacteroides*, *Prevotella*, and *Sutterella* played important roles in glutathione metabolism (Figure 3). However, it was interesting that the relative abundance of these three genera in the feces of T group decreased (Figures 2E,G,H). Experiments have also shown that bacteria used histidine to produce histamine and to prevent the translocation of intestinal bacteria (Snell et al., 1988). In our results, the serum histidine levels in the CRC patients were significantly decreased, which might be due to the decrease in the absorption of histidine into the blood circulation by intestinal flora (Supplementary Figure 2B). Studies had also found that the level of aspartic acid in cancer tissues of the stomach and colon was significantly higher than that in the normal full-thickness or mucosal layer (Okada et al., 1993). This was consistent with our results. The aspartic acid level in CRC tissues was significantly higher than that in the adjacent colonic tissues (Supplementary Figure 2B). In the tumor microenvironment, *Fn* can use amino acids and peptides as nutritional sources without competing for glucose, the substrate of choice for tumor metabolism (Vander Heiden et al., 2009). Therefore, such a nutritional environment not only satisfies the survival conditions of *Fn*, but also facilitates the growth of tumor cells.

Overall, in this study, a variety of assays were used to reveal the association between intestinal flora and metabolites in CRC patients. The distribution of metabolites in various specimens supports the reliability of the data. The metabolites in serum and tissues of patients and healthy individuals were detected by NMR. The metabolite profiles in the tissues were detected using targeted mass spectrometry. Both targeted and non-targeted mass spectrometry were used to detect metabolites in mouse feces. However, due to the small sample size and limited patient specimens, multiple methods could not be performed at the same time. Thus, specimens from different patients were selected for multiple methods. Another limitation of this study is that dietary data were not collected for the CRC patients.

## Conclusion

*Fn* could promote the progression of CRC by affecting the distribution of intestinal flora. Intestinal flora affected the apoptosis of CRC cells through metabolites such as lactic acid and propionic acid. Intestinal flora also could regulate amino acid metabolism. Hence, it is important to explore the role of intestinal flora and their metabolites in the occurrence and development of CRC. This may help to find more effective newer therapeutic targets for treating CRC.

## Data Availability Statement

The datasets presented in this study can be found in online repositories. The names of the repository/repositories and accession number(s) can be found below: National Center for Biotechnology Information (NCBI) BioProject, https://www.ncbi.nlm.nih.gov/bioproject/, PRJNA799322, PRJNA799208, and PRJNA799246 and National Genomics Data Center (NGDC) China National Center for Bioinformation (CNCB)/Beijing Institute of Genomics (BIG), Chinese Academy of Sciences (CAS) Open Archive for Miscellaneous Data (OMIX), https://ngdc.cncb.ac.cn/omix/, PRJCA008313.

## Ethics Statement

The studies involving human participants were reviewed and approved by the Ethics Committee of the First Affiliated Hospital of Wenzhou Medical University. The patients/participants provided their written informed consent to participate in this study. The animal study was reviewed and approved by the Institutional Animal Committee of Wenzhou Medical University.

## Author Contributions

XL and YL: study concept and design. LJ and FJ: specimen collection. TY, SY, XF, and LL: analysis and interpretation of data and statistical analysis. TY, XF, and SY: animal experiments. XL and YL: drafting the manuscript. CL and HG: NMR-based metabolomics experiment. All authors contributed to the article and approved the submitted version.

## Conflict of Interest

The authors declare that the research was conducted in the absence of any commercial or financial relationships that could be construed as a potential conflict of interest.

## Publisher’s Note

All claims expressed in this article are solely those of the authors and do not necessarily represent those of their affiliated organizations, or those of the publisher, the editors and the reviewers. Any product that may be evaluated in this article, or claim that may be made by its manufacturer, is not guaranteed or endorsed by the publisher.

## Supplementary Material

Supplementary Figure 1 A model diagram of animal experiment design.

Supplementary Figure 2 KEGG pathways and metabolite analysis of fecal samples of CRC patients and mice given *Fn* gavage. **(A)** Principal component analysis (PCA) of the OTU and amino acid composition in female vs. male, respectively. **(B)** Histidine in serum of CRC patients and healthy subjects, ^***^*P* < 0.001; **(C)** correlation analysis heat map of bacteria and KEGG pathways in Con, *Fn* and *Fn* + AOM groups; **(D)** aspartate in tissues of CRC patients based on NMR, ^#^*P* < 0.05 vs. control, ^**^*P* < 0.01.
