# Supplementary figures and images for: Fusobacterium nucleatum Affects Cell Apoptosis by Regulating Intestinal Flora and Metabolites to Promote the Development of Colorectal Cancer
Source: Front Microbiol. 2022 Mar 18;13:841157. doi: 10.3389/fmicb.2022.841157 (PMC8971960; doi:10.3389/fmicb.2022.841157)

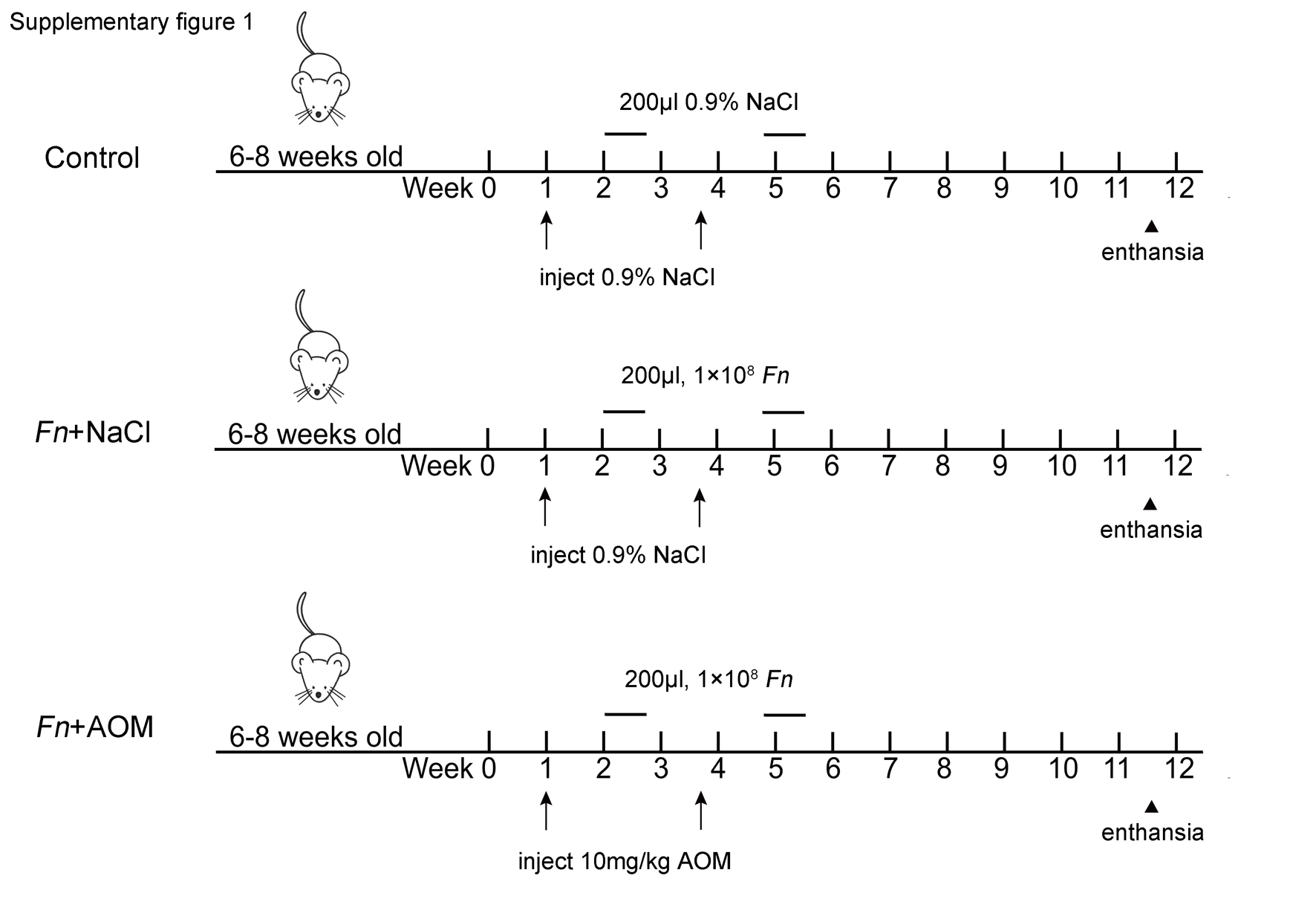

Supplement: Supplementary Figure 1 — A model diagram of animal experiment design. [file Image_1.TIF]

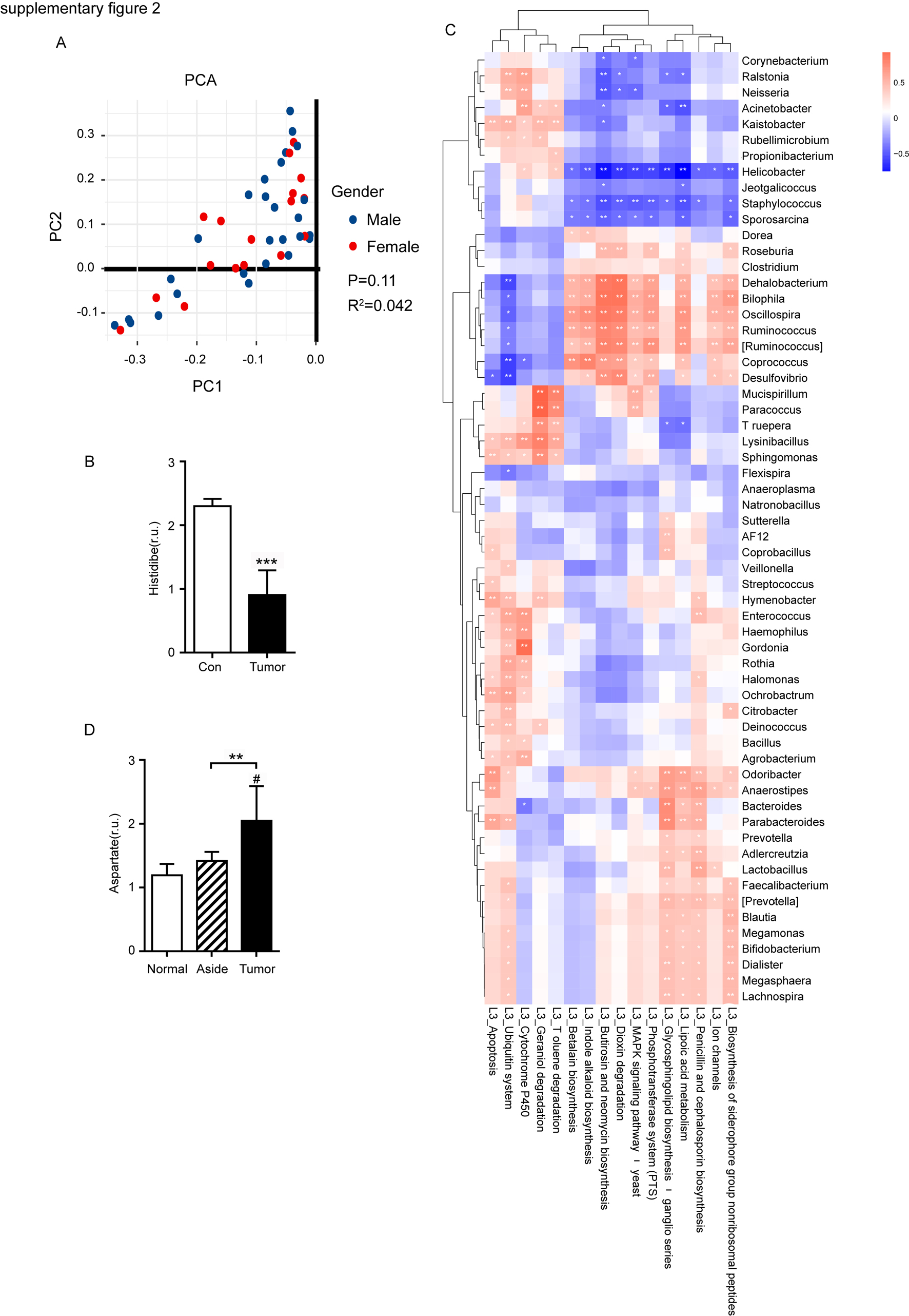

Supplement: Supplementary Figure 2 — KEGG pathways and metabolite analysis of fecal samples of CRC patients and mice given Fn gavage. (A) Principal component analysis (PCA) of the OTU and amino acid composition in female vs. male, respectively. (B) Histidine in serum of CRC patients and healthy subjects, ***P < 0.001; (C) correlation analysis heat map of bacteria and KEGG pathways in Con, Fn and Fn + AOM groups; (D) aspartate in tissues of CRC patients based on NMR, #P < 0.05 vs. control, **P < 0.01. [file Image_2.jpeg]
